# Efficacy between Conventional Laparoscopy and Robotic Surgery in Mexican Patients with Endometriosis: A Comparative Study

**DOI:** 10.3390/jcm13123576

**Published:** 2024-06-18

**Authors:** Cindy Bandala, Juan Pablo Cifuentes-Chacón, Alfredo Cortes-Vázquez, Rodrigo Ruz-Barros, Leonardo Garrocho-Hernández, Alfredo Cortes-Algara

**Affiliations:** 1 Escuela Superior de Medicina, Instituto Politécnico Nacional, Mexico City 07738, Mexico; crodriguezba@ipn.mx (C.B.); alfredo.cortes.vazquez@gmail.com (A.C.-V.); lgarrochoh1601@alumno.ipn.mx (L.G.-H.); 2 Departamento de Laparoscopia y Cirugía Robótica, Centro Médico Nacional “20 de Noviembre”, Instituto de Seguridad y Servicios Sociales de los Trabajadores del Estado, Mexico City 03100, Mexico; jaanpool79@gmail.com (J.P.C.-C.); roemruba@gmail.com (R.R.-B.); 3 UniReproMX Fertility Centre, Mexico City 06700, Mexico; 4 Instituto Materno Infantil del Estado de México, Toluca de Lerdo 50170, Mexico

**Keywords:** conventional laparoscopy, robot-assisted laparoscopy, endometriosis

## Abstract

*Background*. Surgical management of endometriosis is essential, and deep endometriosis involves the invasion of endometrial tissue into other organs such as the bladder, ureters, and rectum. In Latin American countries, significant expertise has been achieved in conventional laparoscopy (CL); however, there is less experience in robot-assisted laparoscopy (RAL) because of the high cost of this technique. For this reason, studies comparing CL and RAL for the treatment of deep endometriosis in patients are scarce, making this study the first to share the experience of Mexican patients. *Aim*. The efficacy of CL vs. RAL in the management of deep endometriosis in Mexican patients was compared. *Materials and Methods*. We performed a retrospective and comparative study. We considered all patients treated with minimally invasive surgery for deep endometriosis between 2015 and 2023. *Results*. A total of 93 patients were included; 56 patients were treated with CL, and 37 patients were treated with RAL. A significant difference (*p* < 0.05) was observed in the postoperative length of stay, which was longer in patients treated with CL compared with those treated with RAL. Additionally, postoperative pain was less frequent in patients treated with RAL than in those treated with CL (*p* < 0.05). We did not observe a significant difference in operative time, blood loss, or perioperative complications between the two surgical techniques (*p* < 0.05). *Conclusions*. CL and RAL are effective methods for managing endometriosis in Mexican patients; however, RAL is beneficial for the treatment of deep endometriosis because patients experience postoperative pain less frequently than CL patients and have a shorter postoperative length of stay.

## 1. Background

Endometriosis is a benign gynecological disease defined by the presence of endometrial glands and stroma outside the uterine cavity; it affects approximately 10% of women of reproductive age [1]. According to data published by the World Health Organization, 190 million women around the globe are diagnosed with endometriosis. Endometriosis is associated with severe pelvic pain that affects quality of life and fertility [2,3]. The diagnostic suspicion is based on classic symptoms, including dyspareunia, dysmenorrhea, dysuria, dyschezia, and infertility [4]. This disease is often classified according to its location, histopathology on the peritoneal surface, presence of ovarian cysts, and deep infiltration [5]. Deep endometriosis is a more difficult endometriotic entity to treat. Surgical management is essential when the lesions are symptomatic and the functions of the affected organs, such as the intestine, bladder, and ovary, are altered [6]. Different methods have been proposed to classify the severity of endometriosis, one of which is the ENZIAN classification. This method is based on the morphological classification of deep endometriosis that can affect compartments such as the vagina, bladder, ureter, intestine, uterine adenomyosis, and even other extragenital locations [7]. The analysis carried out by this classification considers the disease in terms of the size of the deep endometriosis lesion (<5 mm) as well as the location and involvement of the tissues. The pelvis was divided into three compartments, which allows the surgeon to classify the severity of deep endometriosis as follows: grade 1 invasion <1 cm, grade 2 invasion 1–3 cm, and grade 3 invasion >3 cm. The ENZIAN classification is strongly correlated with surgical complexity and complication rates [8]. The management of endometriosis includes psychological, nutritional, medical, and surgical interventions [9]. Treatment decisions are based on demographic criteria (such as age) and clinical criteria (symptom severity, localization and extension, multiple foci of endometrial tissue, and fertility status) [10]. The primary goal of surgery for endometriosis is to relieve symptoms through anatomical and functional restoration by completely resecting endometrial tissue [11].

Minimally invasive surgery, which has demonstrated superior efficiency, has replaced conventional abdominal techniques, and is now considered the technique of choice for surgically treated endometriosis [6,12]. However, conventional laparoscopy (CL) is associated with several challenges, including a long learning curve, long surgical time, surgeon fatigue, a two-dimensional view, the magnification of intraoperative tremors by surgeons, and a limited range of movements, which have restricted the use of this surgical approach [11]. The advantages of robotic-assisted laparoscopy (RAL) include precise incisions, an improved view of the surgical site, an increased range of motion, improved stability by eliminating hand tremors, reduced fatigue, and a shorter learning curve than CL [13]. The advantages of RAL in early-stage endometriosis are not entirely clear [14]. In advanced and deep infiltrating endometriosis, comparative studies have not reported that RAL is better than CL in terms of perioperative outcomes; however, it has been mentioned that RAL has practical advantages over CL from the surgeon’s point of view since better posture implies less deterioration in health, and better maneuverability can lead to more precise treatment [15]. In addition, it has been shown that RAL is more efficient at locating and resecting a greater number of endometriotic foci than CL, which significantly reduces symptoms and recurrence [16]. However, comparative studies of both surgical techniques are controversial, as the results largely depend on the surgeon’s expertise and the affected organ [17].

Currently, in Mexico, the FDA (U.S. Food and Drug Administration) and SSA (Mexican Ministry of Health, acronym in Spanish) have only approved the Da Vinci surgical system from the Intuitive Surgical (Sunnyvale, CA, USA) robotic platform [18,19]. However, there are few studies demonstrating experience in these surgical techniques in Latin American countries. The reasons may be that RAL implies a high cost for public health services, from the acquisition of specialized equipment and staff training; however, its benefits must be demonstrated in comparison with those of CL so that this cutting-edge therapeutic option can become more common in the management of endometriosis patients in our community. Future studies should focus their efforts on identifying specific indications for RAL, considering the technical advantages and their impact on the objective improvement of patients’ symptoms and quality of life [20]. The aim of this study was to evaluate the efficacy of RAL for the treatment of Mexican patients with a diagnosis of deep endometriosis and compare it to that of CL in a high-volume medical center.

## 2. Methodology

### 2.1. Trial Oversight and Patients

We conducted a retrospective and comparative study at the Centro Médico Nacional 20 de Noviembre in Mexico City, Mexico. The Ethics Committee of this hospital approved our study (reference number: 07-182.2023, date: 10 October 2023). All patients who were diagnosed with endometriosis and treated with minimally invasive surgery (CL or RAL) between March 2015 and August 2023 were included. The type of surgery was determined by the hospital’s surgical orientation committee. In cases of endometriosis, surgeons from general surgery, coloproctology, urology, and gynecology were involved. The expert committee considered comorbidities and abdominal surgical history as patients had at least one previous surgery, ultrasound, or MRI. For RAL, patients considered overweight/obese, who had more than two comorbidities, or who had undergone more than two previous abdominal surgeries were included. The exclusion criterion for RAL was that the robot or supplies were not available since it is a public hospital and depends on the annual budget. All patients received the same preoperative pharmacological prophylaxis and general anesthesia. Surgical indications were determined in relation to the patient’s quality of life. All patients were referred for pain, infertility, abnormal uterine bleeding, abdominal distension, urinary and rectal symptoms, and the presence of endometriotic cysts were considered.

### 2.2. Trial Procedures

#### 2.2.1. Surgical Technique Description and Surgeon Experience

The interventions were performed by the surgeons mentioned in this article (C.-A.A. and R.-B.R.), both experienced in CL and RAL, with the assistance of residents or fellows. At the beginning of the study period, in 2015, the surgeons’ experience in CL was 21 and 4 years, respectively, and they had 1 year of experience in RAL.

CL was performed with a Gimmi brand laparoscopy tower and Erbe advanced bipolar energy. An 11 mm umbilical optical trocar and three 5 mm trocars were applied. In most cases, CO_2_ pneumoperitoneum was induced with a Veres needle at Palmer’s point. In all cases, it began with inspection and, if necessary, adhesionysis. Subsequently, resection of endometriotic lesions was carried out via cold cutting depending on the affected organ.

RAL was performed using the da Vinci Surgical System Si (Intuitive Surgical) using up to four ports as needed. The pneumoperitoneum was generated with CO_2_ with a Veres needle at Palmer’s point, following the established norms of 20-10-5 cm, and the robotic trocars were placed using 3 arms and a 5 mm trocar. In the same way as CL, the initial evaluation was carried out and depended on the involvement, adhesiolysis, and resection of the lesions [21].

Surgeons specializing in urology, coloproctology, and gastrointestinal surgery were incorporated into the team depending on the affected organs.

#### 2.2.2. Outcomes

We considered operative time, blood loss, and postoperative length of stay in the hospital as principal outcomes, while postoperative pain and surgical complications were considered secondary outcomes. Age, body mass index (BMI), comorbidities, and the Charlson Comorbidity Index (which predicts long-term mortality in different clinical populations, including surgical patients) [22] were considered. The ENZIAN classification, previous medical treatment, and extrauterine location of endometrial lesions were used to describe the general conditions of the patients included in this study in relation to the type of intervention.

#### 2.2.3. Postoperative Follow-Up

Immediate postoperative pain control was achieved with 1 g of intravenous paracetamol. When the oral route was tolerated, 500 mg of oral paracetamol was administered every 8 h for 3 days. The patient’s discharge criteria were oral tolerance, adequate pain control, diuresis with spontaneous urination (the urinary catheter was removed after 12 h), absence of abdominal alarm signs, and ambulation. It was recommended that patients who experienced abdominal pain, bleeding from the surgical site, fever, or pain immediately visit the emergency room and notify their treating doctor. Patients who progressed favorably were scheduled 7 days after surgery for suture removal, and the healing process was reviewed. After one-week, medical treatment with Dienogest and estradiol or Mirena^®^ was proposed. Subsequent follow-up was monthly during the first 6 months.

### 2.3. Statistical Analysis

The analysis of the data distribution was performed using the Kolmogorov–Smirnov test. Comparisons of frequencies were performed with the chi-squared test or Fisher’s exact test, and we applied the Mann–Whitney U test and Spearman’s rank correlation coefficient. Analyses were performed with SPSS software, version 19 (IBM Corp. Released 2015. IBM SPSS Statistics for Windows, version 19.0. Armonk, NY, USA: IBM Corp.). A *p*-value ≤ 0.05 was considered to indicate statistical significance.

## 3. Results

Ninety-three patients diagnosed with endometriosis who underwent minimally invasive surgical management were included. Two groups were formed according to the type of surgical treatment as follows: 60.2% (56 patients) were treated with CL and 39.8% (37 patients) were treated with RAL. BMI was significantly greater in patients in the RAL group than in those in the CL group (*p* = 0.0001). The type of comorbidity and the Charlson Comorbidity Index were similar in both groups (*p* > 0.05) (Table 1).

Table 2 shows the ENZIAN classification and the history of previous conservative management for endometriosis, with similar frequencies for both types of surgery (*p* > 0.05). The extrauterine location of the endometrial tissue implants differed according to the type of surgery (*p* < 0.05), with multiple foci being more frequent in patients treated with RAL than in those treated with CL.

Table 3 shows the perioperative parameters, postoperative pain, and postoperative complications. There was no significant difference in operative time, although it was longer in the RAL group (181.83 ± 91.12 min) compared with the CL group (146 ± 67.15 min). No significant difference was observed in blood loss, but patients treated with RAL bled less (96.75 ± 124.41 mL) than those treated with CL (113.66 ± 136.66 mL). Patients treated with CL required more days of hospitalization compared with those treated with RAL, and this difference was significant (*p* < 0.05). Postoperative pain was significantly more common in patients treated with CL than in those treated with RAL (*p* < 0.05). In terms of perioperative complications, only one patient in the CL group required conversion to abdominal surgery because of deep endometriosis involving the left ureter. Three patients in the CL group required hand assistance because of deep endometriosis with extrapelvic involvement, primarily affecting the bladder, appendix, and diaphragm. In the RAL group, three patients experienced surgical complications as follows: one with deep endometriosis experienced a right ureter injury, and two with extrapelvic endometriosis had a bladder injury. No statistically significant differences (*p* > 0.05) were observed between the groups regarding surgical complications.

Additionally, we analyzed the correlation between perioperative and postoperative parameters with factors that could influence the results. We observed that in both groups, the operative time was significantly correlated with blood loss (CL: Rho = 0.70, *p* = 0.0001; RAL: Rho = 0.71, *p* = 0.0001) and hospitalization length (CL: Rho = 0.40, *p* = 0.0001; RAL: Rho = 0.50, *p* = 0.002); however, it was not related to the ENZIAN classification in either of the two groups (CL: *p* = 0.39, RAL: *p* = 0.06). Additionally, we found that the Charlson Comorbidity Index was not significantly correlated (CL: *p* = 0.90, RAL: *p* = 0.07). We also found that hospitalization length was correlated with blood loss in both groups (CL: Rho = 0.39, *p* = 0.003; RAL: Rho = 0.46, *p* = 0.004) and with BMI only in the CL group (Rho = 0.36, *p* = 0.006).

## 4. Discussion

Minimally invasive surgery for the management of endometriosis has significant benefits, with lower rates of surgical complications, infection, and postoperative pain, and shorter hospital stays than open surgery [15,17]. Currently, CL is used more frequently than RAL in the management of endometriosis because it is a more feasible technique because of the availability of equipment and staff. However, RAL is gaining importance since studies such as ours are demonstrating benefits that surpass CL in patients with advanced and deep infiltrating endometriosis. CL has certain limitations, including a two-dimensional (2D) operating field, limited reach and freedom of movement, ergonomic challenges for the surgeon, and hand fatigue [6]. RAL employs 3D technology, facilitating better visualization of the surgical site and instrumentation with seven degrees of freedom, providing reduced manipulation of tremors, and reducing work fatigue. Likewise, it shortens the learning curve compared with laparoscopic surgery [17,23,24]. Results in the intraoperative and postoperative outcomes between RAL and CL are contradictory and questionable in relation to the concept of cost–benefit [25]; however, it has been shown that although RAL implies a 20% higher cost compared with CL, the benefit is greater in cases of deep endometriosis, especially in implants in the colon and rectum [20]. More studies are required around this fact.

In our study, we evaluated patients with ENZIAN classification grades 1 to 3, which indicates deep endometriosis, unlike many reports carried out in the United States and European countries that used the American Society for Reproductive Medicine classification (rASRM score) [24,26,27,28]. In this sense, Keckstein and Hudelist proposed that the rASRM score has greater clinical acceptance and is more useful in cases of infertility secondary to endometriosis, while the ENZIAN classification has greater usefulness in deep endometriosis and a greater relationship with complication rates [8]. For these reasons, we use this classification in our study.

Many patients had multiple endometriotic foci and suffered metabolic diseases in addition to being overweight. In deep endometriosis, especially those that require extensive resection, such as deep infiltrative endometriosis, generalized peritoneal implantation, and involvement of the urinary and intestinal tract, a greater benefit has been demonstrated with RAL [14,20,29,30] since it allows better visualization and detection of endometriotic lesions in comparison with CL, as well as a lower risk of complications [16,17,22,24].

Deep endometriosis with multiple foci of intrauterine and extrauterine location is one of the main causes of deterioration in the quality of life of patients. It implies a previous history of therapeutic failure in both pharmacological and surgical procedures [12,20,24,26,31,32,33]. We verified this in our population, as all the patients had at least one previous surgery and the majority had more than two sites of implantation of endometrial tissue located outside the uterus, which was more frequent in the patients treated with RAL [27].

The perioperative outcomes of our study revealed that there was no difference in blood loss or operative time between patients treated with RAL and those treated with CL; this finding coincides with what was reported by different researchers [21,26,31] but differs from what was reported by Ferrier et al., Crestani et al., Volodarsky-Perel et al., Nezhat et al., Moon et al., and Le Gac et al., who observed a greater operative time with RAL than with CL [27,28,30,32,33,34]. An analysis of retrospective studies reported in recent years indicated a minimum operative time for RAL of 106 min [21] and the longest of 250 min [28], while for CL, the minimum time reported was 76 min [33], and the longest was 225 min [31]. Our results showed times considered within these ranges. Operative time can be related not only to the technique and expertise of the surgeon but also to the conditions of the patients. Its relationship with the complexity of the location of endometriotic lesions has been reported based on the ENZIAN classification and extrauterine location [35,36]; however, this finding differs from our results. On the other hand, we observed that operative time is related to blood loss and hospitalization length, which indicates that operative time depends on the complexity of the surgery and the condition of the patients. Regarding blood loss, recent studies report a minimum of 100 mL [21] and a maximum of 184 mL [26] in patients treated with RAL and a minimum of 43.8 mL [21] and a maximum of 188 mL [27] in patients treated with CL. Our study showed a blood loss of less than 100 mL in patients treated with RAL and 113 mL in those treated with CL.

Regarding the days of in-hospital stay, in our study, we observed a significant difference, with patients treated with RAL requiring fewer days than patients treated with CL. This finding also differs from what was reported by Nezhat et al., who observed a stay longer than 24 h in patients treated with RAL compared with CL [32], while other studies found no difference in this parameter [26,27,33,34]. The most recent studies demonstrated a minimum in-hospital stay of 1 day [32] and a maximum of 8 days [26,34] for patients treated with RAL and a minimum of 2 days and a maximum of 8 days for patients treated with CL. Our results were similar to the minimum values in both surgical procedures.

Our results revealed that patients treated with RAL had a lower frequency of postoperative pain than patients treated with CL, which differs from what has been reported in other studies [26]. On the other hand, we did not observe a difference in complications between the two surgical techniques, which is consistent with the findings of others [21,26,27,31,34] with a minimum of conversion to laparotomy in both techniques, similar to our results.

Finally, an important factor related to the variability in the results of perioperative parameters is the surgeon’s experience not only in CL but also in RAL. It has been shown that operative time decreases in relation to the increase in surgeries performed [37], as well as the condition of the patients and the severity of endometriosis [24,26,31], as mentioned before. Further prospective studies comparing laparoscopy to the new robotic systems are needed to address the controversial results that have been reported [38,39,40].

The limitations of this study include its retrospective and non-randomized design, and our selection criteria for choosing the surgery, which were mainly related to the severity of endometriosis and the BMI of the patient. In addition, the outcomes were only measured once, and the postoperative outcomes were not followed up in the long term. Furthermore, this was a single-center study, and the maintenance costs of the robot, and the cost of the instrumentation used in each robotic case compared to analogous laparoscopic interventions were not evaluated in this study; however, this type of investigation is required to contemplate the vision of resource optimization. This study has implications for our health system, demonstrating the benefits of RAL since it allows costs to be reduced because patients require fewer days of hospitalization.

## 5. Conclusions

Our study demonstrated that in the management of deep endometriosis, RAL reduces the length of postoperative stay, and patients treated with this surgical technique experience postoperative pain less frequently than patients treated with CL in a tertiary hospital in Mexico City. The decision to use RAL or CL for patients with endometriosis must be individualized considering the advantages and disadvantages of each of the surgical techniques, and it must also be supported by the surgeon’s experience. Therefore, the objectives of surgical treatment are symptom relief, pain reduction, and patient safety, avoiding complications and minimizing endometrial tissue implants to avoid recurrence. We believe that RAL should be used in the future. We must demonstrate the benefits of this surgical technique so that more equipment can be acquired, and more surgeons can be trained in RAL with the purpose that this technology can benefit a greater number of Latin American patients with endometriosis. However, further studies with prospective designs that explore outcomes not only perioperatively but also postoperatively in the short and long term are needed.

## Figures and Tables

**Table 1:** Clinical and demographic features.

	CL (*n* = 56)	RAL (*n* = 37)	*p*-Value
Age (years) mean ± SD	35.8 ± 8.43	47.27 ± 8.37	0.0001
BMI (kg/m^2^) mean ± SD	25.45 ± 0.65	29.82 ± 0.88	0.0001
Comorbidities	39.3% (22)	48.6% (18)	0.37
Type of comorbidities			
Metabolic	59.1% (13)	83.3% (15)	0.18
Cardiovascular	4.5% (1)	5.6% (1)	
Autoimmune	36.4% (8)	11.1% (2)	
Charlson Comorbidity Index			
0	60.7% (34)	56.8% (21)	0.23
1	19.6% (11)	32.4% (12)	
2	8.9% (5)	10.8% (4)	
3	7.1% (4)	0	
4	3.6% (2)	0	

**Table 2:** Gynecological features related to endometriosis.

	CL (*n* = 56)	RAL (*n* = 37)	*p*-Value
Prior medical treatment	57.1% (32)	43.2% (16)	0.18
ENZIAN classification			
Grade 1	16.1% (9)	18.9% (7)	0.38
Grade 2	41.1% (23)	27% (10)	
Grade 3	42.8% (24)	54.1% (20)	
Extrauterine location			
Peritoneal	46.5% (26)	29.7% (11)	0.009
Ovarian	1.7% (1)	5.4% (2)	
Adhesions	7.1% (4)	0	
Multiple	44.7% (25)	64.9% (24)	

**Table 3:** Perioperative parameters, postoperative pain, and perioperative complications.

	CL (*n* = 56) Median [Mean Range]	RAL (*n* = 37) Median [Mean Range]	Mann–Whitney U	*p*-Value
Operative time (min)	125 [43.66]	170 [52.05]	849	0.14
Blood loss (ml)	50 [46.80]	50 [47.30]	1025	0.93
Hospitalization length (days)	2 [51.88]	1 [39.61]	762	0.01
Postoperative pain	76.7% (43)	40.5% (15)		0.0001 ^&^
Complications	7.1% (4)	8.1% (3)		0.86 ^#^

^&^ Chi square test, ^#^ Fisher’s exact test.

## Data Availability

The data presented in this study are available on request from the corresponding author due to personal and clinical data of the patients are included and the ethics committee requested a confidentiality letter for the management of this data.
